# Prognostic Significance of Nuclear Survivin Expression in Resected Adenoid Cystic Carcinoma of the Head and Neck

**DOI:** 10.1186/1758-3284-2-30

**Published:** 2010-10-30

**Authors:** Yoon Ho Ko, Sang-Young Roh, Hye Sung Won, Eun Kyoung Jeon, Sook Hee Hong, Myung Ah Lee, Jin Hyoung Kang, Young Seon Hong, Min Sik Kim, Chan-Kwon Jung

**Affiliations:** 1 Division of Oncology, Department of Internal Medicine, Uijeongbu St. Mary's Hospital, Catholic University, Gyeonggi-do, South Korea; 2 Division of Oncology, Department of Internal Medicine, Seoul St. Mary's hospital, Catholic University, Seoul, South Korea; 3 Department of Otolaryngology-Head and Neck, Seoul St. Mary's hospital, Catholic University, Seoul, South Korea; 4 Department of Hospital Pathology, Seoul St. Mary's hospital, Catholic University, Seoul, South Korea

## Abstract

**Objectives:**

The expression of survivin, an inhibitor of apoptosis, in tumor cells is associated with poor clinical outcome for various cancers. We conducted this study to determine survivin expression in patients with adenoid cystic carcinoma (ACC) of the head and neck and to identify its clinical significance as a prognostic factor.

**Materials and methods:**

We performed immunohistochemical staining for survivin, p53, bcl-2 protein, and Ki-67 in formalin fixed, paraffin-embedded blocks from 37 cases of head and neck ACC. We also reviewed the patients' clinical records to determine the association of staining with clinical course.

**Results:**

Of the 37 cases of head and neck ACC, 31 (83.8%) were positive for cytoplasmic survivin expression, and 23 (62.2%) were positive for nuclear survivin expression. There was a significant association between nuclear survivin expression and bcl-2 (*P *= 0.031). A larger tumor was more commonly a survivin-positive tumor (cytoplasmic survivin, *P *= 0.043; nuclear survivin, *P *= 0.057). Median overall survival (OS) was significantly longer in patients not expressing nuclear survivin (*P *= 0.035). A multivariate analysis revealed that nuclear survivin expression significantly impacted OS (hazard ratio 8.567, *P *= 0.018) in addition to lymph node involvement (hazard ratio 7.704, *P *= 0.016).

**Conclusions:**

The immunohistochemical expression of nuclear survivin has a prognostic impact in patients with head and neck ACC. These results suggest that nuclear survivin expression may be a useful biomarker for predicting prognosis in patients with head and neck ACC who were treated with surgical resection.

## Background

Adenoid cystic carcinoma (ACC) is an uncommon epithelial tumor that constitutes about 10% of all head and neck tumors. Unlike squamous cell head and neck cancer (HNSCC), ACC has been described as a tumor with indolent but persistent and recurrent growth and late onset of metastases, which eventually leads to death [1]. Several studies have identified clinicopathological factors in ACC with an unfavorable effect on survival, including old age, tumor location, advanced stage, solid histological subtype, high grade, major nerve involvement, the presence of perineural invasion, a positive surgical margin, and lymph node metastasis [2,3]. The primary treatment for ACC is surgery, which is usually followed by post-operative radiotherapy. Although systemic chemotherapy has been used for recurrent or metastatic ACC, there is substantial doubt about its effectiveness and whether systemic therapy impacts on the disease course.

Additional predictors of ACC biologic activity might prove helpful for the clinical management of patients and could be a target of molecular therapy. Biologic prognostic factors including KIT, epidermal growth factor receptor, human epidermal growth receptor-2, estrogen and progesterone receptors, proliferating cell nuclear antigen, Ki-67, and the p53, bcl-2 and SOX-4 genes, have been extensively investigated and are candidates for targeted therapy [4]. However, the results from studies on the effectiveness of several molecular targeted therapies for salivary gland ACC have been disappointing. Thus, more studies are needed for current molecular targeted therapy and further research into novel molecular targets is urgently necessary.

Survivin is one of the most cancer-specific proteins identified to date. It belongs to the apoptosis inhibitor gene family, in which the proteins are characterized by a domain of about 70 amino acids, termed baculovirus inhibitor of apoptosis proteins (IAPs) repeat (BIR) [5]. Unlike other IAPs, survivin is small and has only a single N-terminal BIR domain, a long C-terminal alpha-helix coiled region, and forms a stable dimmer in solution. It inhibits apoptosis differently than bcl-2 either by directly or indirectly interfering with caspase-3 and caspase-7 function via its BIR domain. Survivin also counteracts cell death by interfering with caspase-9 processing, the upstream inhibitor in the intrinsic pathway of apoptosis [6]. Furthermore, survivin enhances cell proliferation and promotes angiogenesis. Survivin is expressed during embryonic and fetal development but is undetectable in terminally differentiated normal adult tissue. However, it is re-expressed in transformed cell lines and several human cancer cells at a frequency of 34-100% [7]. High survivin expression is significantly associated with poor clinical outcomes in various cancers [8-13], including HNSCC [12]. Thus, because of its upregulation in malignancy, it has become of great interest as both a tumor diagnostic and prognostic marker, as well as a new substantial biologic target for future anti-cancer therapies [14]. However, survivin expression in patients with head and neck ACC has not been studied. Moreover, the impact of survival on clinicopathological characteristics and prognosis is unknown.

We investigated the degree of proliferative activity using Ki-67 and the expression of other apoptosis related proteins, bcl-2 and p53. Ki-67 is a nuclear antigen expressed mainly in the S and M phases of the cell cycle, and it has been used for estimating the growth fraction in many studies investigating various tumor types and also in a variety of malignant salivary gland tumors [15]. Bcl-2 proteins play a key role in preventing programmed cell death by favoring prolonged survival in normal and neoplastic cells [16]. The bcl-2 oncoprotein is also proving useful as an investigative tool in oral pathology [17]. Similarly, the p53 protein stimulates the transcription of several genes that mediate cell cycle arrest, and this protein initiates apoptosis in response to DNA damage. While the wild-type p53 protein makes tumor differentiation possible, the mutant p53 protein blocks it [18].

Thus, in the present study, we examined survivin expression in surgical specimens from patients with head and neck ACC using tissue microarray and immunohistochemical methods. We also investigated survivin expression in patients with head and neck ACC and its association with other biologic markers and clinical outcomes.

## Methods

### Patients and specimens

This study was approved by the Uijeongbu St. Mary's hospital institutional review board. All of the tissues investigated were obtained from 42 consecutive patients with head and neck ACC who underwent a primary resection between April 1997 and March 2003 at Seoul St. Mary's Hospital, the Catholic University of Korea. Paraffin blocks with the tumor samples were available from 37 patients. The demographic features of these patients are summarized in Tables 1 and 2. The median follow-up time was 83.5 months (range, 8.2-213.9), and the median age of the patients was 53 years (range, 28-75 years). According to the American Joint Committee on Cancer staging criteria, 19 patients (51.4%) had stage I and II disease, and 18 patients (48.6%) had stage III and IV disease. Four patients (10.8%) had positive lymph nodes and 33 (89.2%) had negative lymph nodes. At the end of the follow-up period, 18 patients (48.6%) had died, and the median overall survival time was 164.4 months (95% confidence intervals (CI), 50.830-277.970).

**Table 1:** Baseline clinical and medical characteristics of patients with head and neck adenoid cystic carcinoma.

Characteristics	Total	
	
	No. of patients	%
No. of patients	37	
Age (years), median (range)	53.0 (28 - 75)	
Gender		
Male	14	37.8
Female	23	62.2
Primary site		
Major salivary gland*	14	37.8
Minor salivary gland^†^	23	62.2
Stage		
I	7	18.9
II	12	32.4
III	11	29.7
IV	7	18.9
Lymph node involvement		
Positive	4	10.8
Negative	33	89.2

* submandibular gland, parotid gland, sublingual gland.

^† ^maxillary sinus, nasal cavity, base of tongue, floor of the mouth, external acoustic canal.

**Table 2:** Baseline pathological characteristics of patients with head and neck adenoid cystic carcinoma.

Characteristics	Total	
	
	No. of patients	%
Tumor size (cm) (n = 32)		
≤ 3 cm	14	43.8
> 3 cm	18	56.2
Histological growth pattern		
Tubular	7	18.9
Cribriform	23	62.2
Solid	7	18.9
Histological grade		
Well	6	16.2
Moderately	21	56.8
Poorly	10	27.0
Perineural invasion (n = 34)		
Positive	26	76.5
Negative	8	23.5
Perivascular invasion (n = 35)		
Positive	4	11.4
Negative	31	88.6
Lymphatic invasion (n = 33)		
Positive	11	29.7
Negative	22	66.7

### Construction of the tissue microarray

All archival tissue samples were routinely fixed in formalin and embedded in paraffin wax. Representative tissue areas were marked on standard hematoxylin and eosin stained sections that were cut from the blocks; these corresponding areas were then punched out of the paraffin block using a 2.0-mm punch, and the cores were inserted into a recipient paraffin block. To decrease any error introduced by sampling and to minimize the impact of tissue loss during processing, duplicate tissue cores per specimen were arrayed on a second recipient paraffin block. Sections (5 μm) were cut from the completed array block and transferred to silanized glass slides.

### Immunohistochemistry and analysis

Immunohistochemical staining was performed on 5 μm sections of the tissue microarray blocks using a Lab Vision Autostainer LV-1 (LabVision/Neomarkers, Fremont, CA, USA), according to the manufacturer's protocol. Paraffin sections were mounted on superfrost glass slides, deparaffinized, and rehydrated in a graded ethanol series. The antigen was retrieved with 0.01 M citrate buffer (pH 6.0) by heating the sample in a microwave vacuum histoprocessor (RHS-1, Milestone, Bergamo, Italy) at a controlled final temperature of 121°C for 15 min. Endogenous peroxidase activity was blocked by incubating the slides in 3% hydrogen peroxide in methanol for 10 min. The primary antibodies were diluted in Dako Antibody Diluent (Dako, Carpentaria, CA, USA) with background-reducing components and were used at the following dilutions: survivin (1:1000, polyclonal, Novus, Littleton, CO, USA), p53 (1:100, clone DO-7, monoclonal, Dako), bcl-2 (1:100, clone 124, Dako), and Ki-67 (1:50, clone MIB-1, Dako). The primary antibodies were incubated at room temperature for 30 min and detected using the Envision Plus System (Dako). The immunoreaction was developed with diaminobenzidine (Dako) for 5 min and counterstained with hematoxylin. Results were interpreted by one pathologist (C.K.J.) who was blinded to the specific diagnosis and prognosis for each case. For survivin staining, staining intensities were scored as no staining (0), weak staining (1+), moderate staining (2+), or strong staining (3+). The percentage of staining area was classified as 0, 0%; 1, 1-10%; 2, 11-50%; 3, 51-100%. The intensity and percentage scores were multiplied to give a composite score of 1-9 for each specimen. Composite scores of 1-3 were defined as having low surviving protein expression, and scores of 4-9 were considered to be high expression of survivin. For bcl-2, p53, and Ki-67 staining, tumors were considered to be positive expression if ≥10% of tumor cells were immunostained.

### Statistical Methods

Statistical calculations were performed using the SPSS software package (version 13.0; SPSS, Chicago, IL, USA). Overall survival was measured from the date of diagnosis to the date of death or the last follow-up visit. Survival was derived by the Kaplan-Meier method, and the statistical differences in the cumulative survival curves were evaluated using the log-rank test. Multivariate survival analysis was performed using the Cox proportional hazard model. All variables with a P-value less than 0.2 in the univariate analysis were selected for the multivariate analysis. The immunohistochemical profiles were compared to the clinicopathological parameters using the chi-square and Fisher's exact tests. Survival rates and odds ratios are presented with their 95% confidence interval (CI). Statistical tests were two-sided at the 5% level of significance.

## Results

### Expression of survivin, bcl-2, p53, and Ki-67

**Figure 1:**
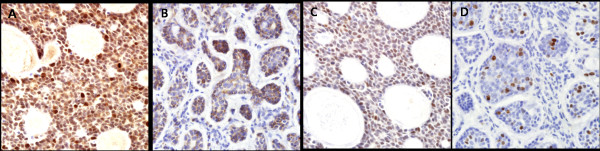
**Immunohistochemical staining for survivin, bcl-2, p53, and Ki-67 in adenoid cystic carcinomas**. (A) Most tumor cells showed diffuse nuclear and cytoplasmic staining for survivin (staining score, 3). (B) Bcl-2 was expressed diffusely in the cytoplasm of tumor cells. The tumor cells showed positive nuclear staining for p53 (C) and Ki-67 (D). Original magnifications ×400, A-D.

Cytoplasmic staining for survivin was observed in 31 of 37 cases (83.8%; Figure 1A), whereas nuclear staining for survivin was observed in 23 cases (62.2%). Bcl-2 was strongly expressed mainly in the cytoplasm and membranes of cancer cells, with 51.4% higher scores (19 of 37; Figure 1B). p53 expression was detected in the cancer cell nuclei in 9 of 37 cases (24.3%). Four cases (10.8%) were positive for Ki-67 immunohistochemical staining. The associations between cytoplasmic/nuclear survivin and bcl-2, p53, or Ki-67 expression are shown in Table 3. Nuclear survivin expression was significantly associated with bcl-2 expression (*P *= 0.031). There was a tendency for an association between cytoplasmic survivin and bcl-2 expression, but the difference was not significant (*P *= 0.078).

**Table 3:** Relationship among clinicopathological factors and marker expression patterns

	C-survivin*			N-survivin^†^		
	Low, n(%)	High, n(%)	P-value	Low, n(%)	High, n(%)	P-value
Histological growth pattern						
Tubular, cribriform	6(100)	24(77.4)	0.255	12 (85.7)	18(78.3)	0.459
Solid	0(0)	7(22.6)		2(14.3)	5(21.7)	
Grade						
Well, moderately	5(83.3)	22(71.0)	0.475	12 (85.7)	15(65.2)	0.164
Poorly	1(16.7)	9(29.0)		2(14.3)	8(34.8)	
Stage						
I, II	4(66.7)	15(48.4)	0.357	10(71.4)	9(39.1)	0.357
III, IV	2(33.3)	16(51.6)		2(28.6)	14(51.6)	
Tumor size						
≤ 3 cm	5(83.3)	9(34.6)	0.043^‡^	8(61.5)	11(42.1)	0.057
> 3 cm	1(16.7)	17(65.4)		5(38.5)	8(57.9)	
Lymph node involvement						
Negative	6(100)	27(87.1)	0.476	6(46.2)	11(87)	0.821
Positive	0(0)	4(10.8)		7(53.8)	3(13)	
Bcl-2						
Negative	5(83.3)	13(41.9)	0.078	10(71.4)	8(34.8)	0.031^‡^
Positive	1(16.7)	18(58.1)		4(28.6)	15(65.2)	
Ki-67						
Negative	6(100)	27(87.1)	0.476	13(92.9)	20(87)	0.509
Positive	0(0)	4(12.9)		1(7.1)	3(13)	
P53						
Negative	5(83.3)	23(74.2)	0.543	12(85.7)	16(69.6)	0.267
Positive	1(16.7)	8(25.8)		2(14.3)	7(30.4)	

* cytoplasmic survivin

^† ^nuclear survivin

^‡ ^statistically significant (P < 0.05)

### Correlation between the biological markers and clinicopathological characteristics

High survivin expression was found more frequently in tumors greater than 3 cm in diameter (cytoplasmic survivin, *P *= 0.043; nuclear survivin, *P *= 0.057; Table 3) than in those smaller than 3 cm in diameter. However, survivin expression was not associated with histological growth pattern or histological grade. Low bcl-2 expression was marginally associated with perineural invasion (*P *= 0.080). There were no significant interactions between bcl-2 expression and any other clinicopathological factors (data not shown).

### Clinical outcome and survivin expression

**Figure 2:**
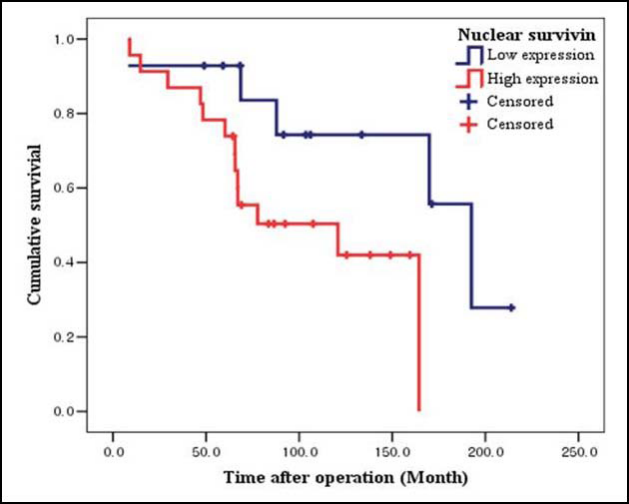
**Kaplan-Meier survival estimate for overall survival of patients with adenoid cystic carcinoma according to survivin expression**.

Table 4 shows the association of patients' characteristics and clinicopathological features with overall survival in the 37 patients analyzed by univariate analysis. The median overall survival time was 164.4 months (95% CI, 50.8-278.0) for all patients, with a median overall survival of 120.8 months (95% CI, 28.6-213.0) months for those with high nuclear survivin expression, and a median overall survival of 192.5 months (95% CI, 157.8-227.2) for those with low nuclear survivin expression. The 71.7-month difference in the overall survival between the above two groups was statistically significant (*P *= 0.035), whereas the median overall survival was 120.8 months (95% CI, 39.6-202.0) for patients with high cytoplasmic survivin expression and was not reached for those with low expression (median duration, 176.8 months; 95% CI, 123.8-239.8). However, the survival difference was statistically significant (*P *= 0.160). The expression of any other markers was not significantly correlated with overall survival. In addition, clinical parameters including TNM stage, lymph node involvement, and tumors greater than 3 cm in diameter were significantly correlated with overall survival (*P *= 0.001, *P *= 0.014, and *P *= 0.036, respectively). The final multivariate analysis is shown in Table 5 and Figure 2. The significant predictors were lymph node involvement and high nuclear survivin expression (*P *= 0.016 and *P *= 0.018). However, high cytoplasmic survivin expression did not achieve a statistically significant level (*P *= 0.734). Other clinicopathological factors were not statistically associated with overall survival.

**Table 4:** Clinicopathological variables affecting overall survival (univariate analysis).

Variable	P-value (chi-square)
Stage (I, II/III, IV)	0.001*
Lymph node metastasis	0.014*
Tumor size (≤ 3 cm/> 3 cm)	0.036*
Growth pattern (tubular, cribriform/solid)	0.806
Tumor grade (well, moderately/poorly)	0.235
Perineural invasion	0.377
Perivascular invasion	0.926
Lymphatic invasion	0.569
C-survivin^† ^(high/low)	0.160
N-survivin^‡ ^(high/low)	0.035*
Bcl-2 (positive/negative)	0.986
Ki-67 (positive/negative)	0.872
p53 (positive/negative)	0.957

* statistically significant (P < 0.05)

^† ^cytoplasmic survivin

^‡ ^nuclear survivin

**Table 5:** Multivariate analysis of the clinicopathological characteristics and four biological factors by overall survival rate.

Characteristics	Hazard ratio	95% CI	p-value
N-survivin*	8.567	1.445-50.783	0.018
Lymph node involvement	7.704	1.468-40.434	0.016

CI, confidence interval.

*nuclear survivin

## Discussion

We examined survivin expression in patients who underwent resection for head and neck ACC. The nuclear expression of survivin, which was more frequently observed in larger tumors, was significantly correlated with unfavorable clinical outcome. To the best of our knowledge, this study is the first to demonstrate a significant correlation between survivin expression and clinical prognosis in patients with resected ACC of the head and neck. These data suggest that nuclear survivin expression aggressively identifies cases of head and neck ACC and, therefore, could influence the decision for therapy at the time of diagnosis.

Several studies have identified clinicopathological factors with an unfavorable effect on survival in ACC, including old age, tumor location, advanced stage, solid histological subtype, high grade, major nerve involvement, the presence of perineural invasion, and the presence of a positive surgical margin [2]. In our previous report, only lymph node involvement was predictive of overall survival [3]. However, using conventional clinopathological criteria, it is difficult to develop an accurate prognosis and treatment response for advanced head and neck ACC. When we analyzed the survival biomarkers in this study, a stepwise Cox analysis showed that nuclear survivin was significantly associated with survival. When we analyzed the survival biomarkers in this study, a stepwise Cox analysis showed that nuclear survivin was significantly associated with survival.

Survivin has been studied as a prognostic marker in various cancers. Patients with survivin-positive tumors have a decreased apoptotic index and worse survival rates than those with survivin-negative tumors. In addition, survivin expression has been correlated with resistance against chemotherapy- and radiotherapy-induced apoptosis and abbreviated patient survival [14]. Previous studies have correlated survivin with an unfavorable clinical outcome in a variety of cancers, including colorectal cancer [8], breast cancer [9], lung cancer [10], esophageal cancer [11], brain tumor [13], soft tissue sarcoma [19], and hematologic malignancies [20].

In the present study, with a median follow-up time of 83.5 months, patients with high nuclear survivin expression had poorer overall survival than those with low nuclear survivin expression (median duration, 120.8 vs. 192.5 months; hazard ratio, 8.567; 95% CI, 1.445- 50.783; *P *= 0.018). Moreover, survivin expression, especially subcellular nuclear localized expression, was a strong independent negative predictor of overall survival. Little data exist on the expression or clinical implications of survivin in head and neck ACC. On the contrary, the clinical significance of survivin expression in HNSCC has been reported for oral [21], oropharyngeal [22], and laryngeal carcinoma [23]. Our results are supported by several reports. Lo Muzio et al. [21], in a series of 110 oral SCC cases, found that patients with low survivin expression had significantly better survival rates than patients with medium and high survivin expression. Presuss et al. [22] showed that nuclear survivin expression was associated with a poor overall survival rate, with an estimated 3-year overall survival probability of 17.3% vs. 87.4% for non-nuclear expression of survivin (p < 0.001) in 73 patients with surgically treated oropharyngeal SCC. Dong et al. [23] examined 102 cases of laryngeal SCC, and found that survivin expression was significantly associated with shorter disease-free and overall survival (hazard ratio, 0.2696; 95% CI, 0.02666-0.85475; *P *0.05).

In contrast, Freier et al. [24] found that high survivin expression was associated with increased 3-year, 5-year, and 10-year overall survival in tumors from 296 patients with advanced oral SCC who were treated with radiotherapy (*P *= 0.005, *P *= 0.004 and *P *= 0.002, respectively). The authors concluded that high survivin expression might be useful for identifying patients with oral SCC who could benefit from radiotherapy. These inter-report differences may be associated with the histological tumor types, different treatment modalities, the various immunohistochemistry protocols and/or antibodies used, or be due to variable criteria applied to annotate a tumor as nuclear- or cytoplasmic-survivin positive. In fact, Freier et al. did not analyze nuclear and cytoplasmic staining to categorize immunostaining independently.

Survivin exists in distinct nuclear and cytoplasmic subcellular pools in human cancer cells [25]. However, the clinical implications for subcellular localization of survivin expression remains controversial. Among the 19 publications relevant to survivin localization in nuclei or cytoplasm in various cancer tissues reviewed by Li et al. [26], 9 showed that survivin expression in cancer cell nuclei was an unfavorable prognostic marker, whereas 5 proposed the opposing notion that nuclear survivin expression represented a favorable prognostic marker. Similarly, overall survivin expression, its discrete intracellular localization, and its implication as a prognostic marker were also analyzed in several HNSCC studies, albeit with opposing results [12,22,27]. In a series of nine patients with laryngeal basaloid squamous cell carcinoma, Marioni et al. [12] found that high nuclear survivin expression is associated with disease recurrence and poor prognosis (*P *= 0.02). Khan et al. [27] did not find a significant correlation between the survivin expression pattern and clinicopathological parameters in patients with oral SCC. Lo Muzio et al. [21] in a series of 110 cases of oral SCC, found a significant correlation between the cytoplasmic survivin expression pattern and poor clinical outcome. Recently, four alternatively splicing transcripts have been identified in a single copy of the survivin gene. In addition to wild-type survivin, four survivin variants (survivin-2A,-2B,-3B and -ΔEx3) are generated [28]. These transcripts may have different subcellular localizations. All transport occurs through the nuclear pore multiprotein complex. Recent convincing experimental data suggest that survivin contains Crm1-dependent nuclear export signals (NES) in the linker region between the BIR domain and the C-terminal alpha helix. Consistent with this finding, the NES-deficient survivin isoforms survivin-ΔEx3 and survivin-2A do not localize predominantly in the cytoplasm, whereas the NES-containing variants survivin-2B and survivin-3B are cytoplasmic [29]. Nuclear survivin is also a subunit of the chromosomal passenger complex, which ensures the correct completion of cytokinesis and is composed of the mitotic kinase aurora-B, borealin, and INCENP [30]. However, their functions in carcinogenesis are largely unknown, and why survivin displays a predominant nuclear localization in some tumors but not in others is unclear. Functionally, one could consider that the nuclear pool of survivin is involved in promoting cell proliferation in most cases, whereas cytoplasmic survivin may participate in controlling cell survival but not cell proliferation. Nuclear survivin may help maintain the integrity of the mitotic spindle in cancer cells [30], and strong nuclear survivin staining may represent an increased number of mitotic events, resulting in poor survival [10]. In many immunohistochemical studies, nuclear survivin expression is an unfavorable factor for prognosis, including prostate cancer [31], rectal cancer [32], esophageal squamous cell carcinoma [11], colorectal carcinoma [8], soft-tissue sarcoma [19], breast cancer [9], laryngeal squamous cell carcinoma [12], hepatocellular carcinoma [33], ovarian carcinoma [34], non-small cell lung carcinoma [10], and glioblastoma [13]. In contrast, few studies have reported immunohistochemical cytoplasmic survivin expression as an unfavorable factor in patients with colorectal cancer [30], pancreatic cancer [35], or oral squamous cell carcinoma [21]. Thus, further investigations are required to clarify the prognostic value of nuclear/cytoplasmic survivin expression.

In this study, nuclear and cytoplasmic survivin was highly expressed in 62.2% and 83.8% of the ACC specimens, respectively. Similarly, in HNSCC, despite the use of variable cut-off values, previous studies have reported that survivin is expressed in 12% to 72% of patients with HNSCC [27,36]. The analysis of survivin and the clinicopathological factors showed a significant association between tissue expression of survivin and tumor size but not lymph node involvement, which has also been observed in previous studies on other cancers, including laryngeal cancer [23]. The correlation between survivin expression and tumor stage or the presence of lymph node metastases in patients with primary HNSCC is still a matter of debate. In Khan et al., there was no significant association between survivin and tumor stage. Considering lymph node metastasis, Marioni et al. [36], in an evaluation of 13 consecutive cases of oral and oropharyngeal SCC with pN+ and 13 cases of pN0, demonstrated that eight patients in the pN+ group were survivin-positive (mean expression 34.7%), compared to five in the pN0 group (12.3%), and this difference was statistically significant (*P *= 0.017). In contrast, Lo Muzio et al. [21], in an analysis of 110 oral SCC cases, reported that there was no significant correlation between survivin expression and the presence of lymph node metastases.

We found that the association between survivin expression and bcl-2 was correlated statistically (nuclear expression, *P *= 0.031; cytoplasmic expression, *P *= 0.078). The bcl-2 oncoprotein is a potent inhibitor of apoptosis and is overexpressed in a wide variety of malignancies, including salivary gland tumors [17]. One of antiapoptotic mechanisms by which bcl-2 may mediate cell cytoprotection independently of cytochrome c release is through increased survivin expression [37]. However, we did not find significant correlations between survivin and p53 or ki-67 expression. Khan et al. [27], in a series of 29 oral SCC cases, observed that about half of the p53-positive oral SCC and premalignant tissues also showed significant survivin positivity. Furthermore, Ki-67 was expressed at a rate of 10.8%, in contrast to that of survivin. This relatively low expression Ki-67 rate may explain the indolent natural course of head and neck ACC [38]. Ki-67 values greater than 10% have been demonstrated to be the most significant indicator of short-term clinical course in ACC [39].

The present study has several limitations. First, it was a relatively small number of patients. Second, we measured expression using only immunohistochemical staining, which has several weak points, including a semiquantitative nature, tissue aging effects, the staining technique, the enzyme antibody used, and single observer bias.

In conclusion, the present study demonstrated that nuclear survivin expression has clinicopathological implications in patients with head and neck ACC. We expect that our data on the clinical implications of survivin will provide new insights into the management of head and neck ACC. Survivin may be an ideal target for therapy to improve the prognosis of patients with head and neck ACC. Further investigation is necessary to clarify and understand the roles of survivin in patients with head and neck ACC.

## Competing interests

The authors declare that they have no competing interests.

## Authors' contributions

YHK: Study design, statistical analysis, and preparation of the article for publication. CKJ: Implementation of the immunohistochemical procedures, immunohistochemical interpretation, histological examination and grading, immunohistochemical interpretive calibration and peer reviewing the final draft. SYR, HSW, EUK, SHH, MAL, JHK, YSH: Performing the chemotherapy and management and peer reviewing the final draft. MSK: Performing the surgical operation. All authors read and approved the final manuscript.
